# ERDHEIM‐Chester Disease: Two Fatal Cases With Unusual Presentations

**DOI:** 10.1002/ccr3.72798

**Published:** 2026-05-26

**Authors:** Ribero‐Vargas Daniel Andres, C. Álvarez Payares José, Murillo‐Pérez Juliana

**Affiliations:** ^1^ Internal Medicine Clínica CES Medellín Colombia; ^2^ Hematology Clinica de Oncologia Astorga Medellín Colombia; ^3^ Internal Medicine Pablo Tobón Uribe Hospital Medellín Colombia

**Keywords:** Erdheim‐Chester disease, gastrointestinal, hairy kidney sign, histiocytosis, serositis

## Abstract

We present two fatal cases of Erdheim‐Chester disease (ECD) with unusual presentations: one featuring predominant gastrointestinal involvement with steatorrhea, nodular gastroduodenitis, and extensive visceral infiltration, and another with constrictive pericarditis and pachypleuritis. Both cases displayed characteristic imaging findings including the “hairy kidney” sign, “coated aorta” sign, symmetric long bone osteosclerosis, and diffuse FDG‐PET avidity in bone marrow and serosal surfaces. Histopathology confirmed xanthogranulomatous infiltration with foamy histiocytes and Touton giant cells, with immunohistochemistry showing CD68‐positive, CD163‐positive, CD1a‐negative, and S100‐negative cells lacking Birbeck granules, consistent with non‐Langerhans cell histiocytosis. These atypical organ manifestations, when accompanied by characteristic radiological signs, should prompt consideration of ECD in the differential diagnosis of multisystem inflammatory disorders.

## Key Clinical Message

Erdheim‐Chester disease may present with unusual gastrointestinal and serosal manifestations, including chronic diarrhea, serositis, and constrictive pericarditis. These atypical presentations, when combined with characteristic imaging findings such as “hairy kidney” sign and bone involvement, should prompt consideration of this rare non‐Langerhans histiocytosis for timely diagnosis and targeted therapy.

## Graphical abstract

Fluorodeoxyglucose positron emission tomography‐computed tomography (FDG PET‐CT) demonstrated markedly increased metabolic activity in serosal surfaces including pericardium, pleura, and renal fascia, as well as diffuse uptake along vascular walls, particularly the entire aorta.

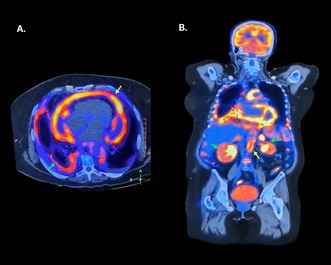

## 1 Introduction

Erdheim‐Chester disease (ECD) is an uncommon systemic form of non‐Langerhans cell histiocytosis of unknown etiology, characterized by xanthogranulomatous infiltration of foamy histiocytes surrounded by fibrosis [1]. ECD may be asymptomatic or present as multisystem disease with potentially fatal manifestations affecting the skeleton, central nervous system, respiratory, cardiovascular, and renal systems, as well as the retroperitoneum and skin, with potentially fatal manifestations [2]. This article reviews two patients presenting with predominant gastrointestinal symptoms and serosal involvement.

## 2 Case Reports

### 2.1 Case 1

#### 2.1.1 Presentation and Clinical Course

A 43‐year‐old male with no significant medical history presented with a 2‐year history of progressive lower extremity edema that evolved to anasarca over 6 months, associated with steatorrhea, intermittent fever, and night sweats. Physical examination revealed an erythematous‐violaceous maculopapular rash on the neck and chest, hepatosplenomegaly, and grade III pitting edema in lower extremities (Figure 1).

**FIGURE 1:**
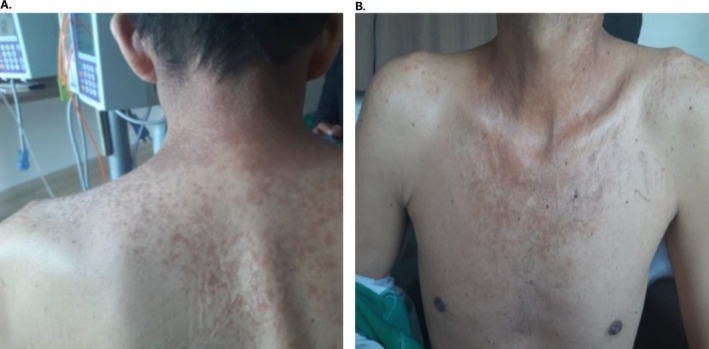
Physical examination findings in Case 1. Erythematous‐violaceous maculopapular rash on posterior neck (A) and anterior chest wall (B), associated with constitutional symptoms and multiorgan involvement.

#### 2.1.2 Diagnostic Workup

Initial laboratory studies documented severe pancytopenia (hemoglobin 7.2 g/dL, white blood cell count 2100/μL, platelet count 68,000/μL), hypoalbuminemia (2.1 g/dL), vitamin B12 deficiency (180 pg/mL), prolonged coagulation times (PT 18.2 s, aPTT 45 s), hypofibrinogenemia (120 mg/dL), and hypocomplementemia (C3 45 mg/dL, C4 8 mg/dL). The initial differential diagnosis included hematologic malignancy, systemic autoimmune disease, chronic infection, and infiltrative disorders.

Chest and abdominal CT performed to evaluate constitutional symptoms and cytopenias showed right pleural effusion and hepatosplenomegaly without lymphadenopathy. Given the gastrointestinal symptoms and hypoalbuminemia, upper endoscopy was performed, revealing antral erythematous gastritis and chronic duodenitis. Ileocolonoscopy with biopsy showed nodular lymphoid hyperplasia in the ileum. Skin lesion biopsy demonstrated granulomatous dermatitis.

FDG PET‐CT revealed extensive increased bone marrow metabolism throughout the entire axial and appendicular skeleton (SUVmax 8.4 in femoral diaphysis, 7.8 in vertebral bodies, 6.9 in ribs). Bone marrow biopsy showed a complex karyotype with three distinct clones (one with Y chromosome gain, one with trisomy 8, and one with trisomy 12) accompanied by chronic non‐necrotizing granulomatous inflammation (Figure 2). The marrow demonstrated extensive replacement of normal architecture by foamy histiocytes.

**FIGURE 2:**
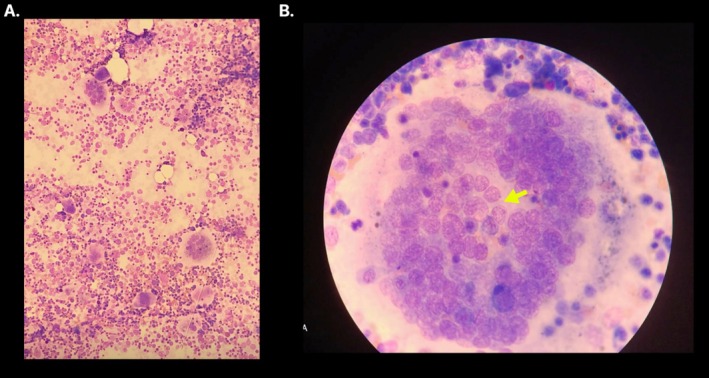
Bone marrow biopsy findings in Case 1. (A) Low‐power view (100×, H&E stain) showing extensive replacement of normal bone marrow architecture by sheets of foamy histiocytes with surrounding fibrosis. (B) High‐power view (400×, H&E stain) demonstrating foamy histiocytes with abundant pale cytoplasm and Touton‐type multinucleated giant cells (arrow) characteristic of Erdheim‐Chester disease. Scale bars: A = 100 μm, B = 50 μm.

#### 2.1.3 Molecular Testing

BRAF V600E mutation testing was not performed due to the patient's rapid clinical deterioration and death before molecular studies could be completed. This limitation precluded targeted therapy consideration.

#### 2.1.4 Outcome

The patient died from multisystem failure within 3 weeks of diagnosis, before receiving targeted therapy. Autopsy findings revealed multiple mononuclear cell accumulations with abundant clear cytoplasm and perivascular infiltrates composed of Touton‐type multinucleated giant cells in dura mater, subcutaneous tissue, tracheal mucosa, lung, pleura, deltoid muscle, endocardium, coronary arteries, liver, and duodenal tissue. Bone marrow showed extensive replacement by foamy histiocytes. Silver methenamine and periodic acid‐Schiff stains excluded fungal infection and amyloidosis.

Electron microscopy revealed macrophages with slightly irregular nuclei and cytoplasm containing multiple phagocytic lysosomes without Birbeck granules. Immunohistochemistry showed strong CD163 positivity confirming histiocytic lineage, but negative for Langerhans cell markers (langerin, CD1a) with only minimal focal S100 positivity (< 5% of cells) and CD68 positivity, confirming non‐Langerhans histiocytosis. The absence of BRAF testing limited definitive prognostic stratification, though the aggressive clinical course and multisystem involvement suggested poor‐risk disease biology.

### 2.2 Case 2

#### 2.2.1 Presentation and Clinical Course

A 64‐year‐old woman with no relevant medical history presented with 2 years of progressive dyspnea, initially on moderate exertion but advancing to dyspnea at rest over the preceding 6 months. Associated symptoms included orthopnea, dry cough, fatigue, decreased appetite, and progressive lower extremity edema. Physical examination revealed jugular venous distension, bibasilar crackles, grade II pitting edema in lower extremities, and xanthelasmas on eyelids and neck.

#### 2.2.2 Diagnostic Workup

Initial transthoracic echocardiography revealed global pericardial effusion (largest dimension 18 mm) with right pleural effusion, without obvious valvular abnormalities or reduced ejection fraction. The clinical differential diagnosis included constrictive pericarditis, restrictive cardiomyopathy, autoimmune pericardial disease, tuberculous pericarditis, and malignancy. Pericardiocentesis drained 480 mL of hemorrhagic fluid; cytology showed reactive mesothelial cells without malignant cells, and microbiological cultures (bacterial, fungal, mycobacterial) were negative.

Contrast‐enhanced thoracoabdominal CT demonstrated severe pachypleuritis (pleural thickness up to 8 mm), pachypericarditis (pericardial thickness 9 mm), and excessive soft tissue surrounding and encasing the aorta from the aortic arch through the abdominal aorta (perivascular rind thickness 6–12 mm), creating the characteristic “coated aorta” sign. Bilateral perinephric soft tissue infiltration (thickness 8–15 mm) produced the “hairy kidney” appearance. Additional findings included adrenal gland hypertrophy and hepatomegaly (Figure 3).

**FIGURE 3:**
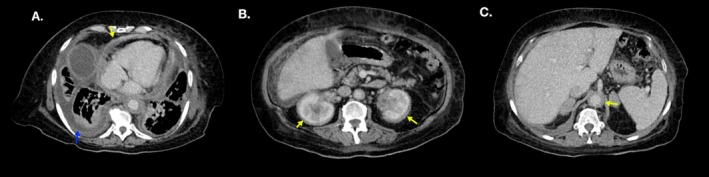
Contrast‐enhanced chest and abdominal computed tomography (CT), axial views, in Case 2. (A) Mediastinal window showing pachypericarditis (pericardial thickness 9 mm, yellow arrow) with pericardial effusion and pachypleuritis (pleural thickness 8 mm, blue arrow). (B) Soft tissue window at renal level demonstrating bilateral perinephric soft tissue infiltration (thickness 8–15 mm, yellow arrows) creating the characteristic “hairy kidney” sign. (C) Soft tissue window showing hepatomegaly (craniocaudal span 18 cm) and circumferential soft tissue encasing the abdominal aorta (perivascular rind thickness 6–12 mm, yellow arrow), the “coated aorta” sign suggestive of Erdheim‐Chester disease.

Cardiac MRI confirmed severe diffuse pericardial thickening (maximum thickness 11 mm) with septated pericardial effusion, respirophasic ventricular septal shift consistent with ventricular interdependence, and severe diffuse aortic wall thickening (wall thickness 8–14 mm) throughout all visualized segments with intense late gadolinium enhancement (Figure 4).

**FIGURE 4:**
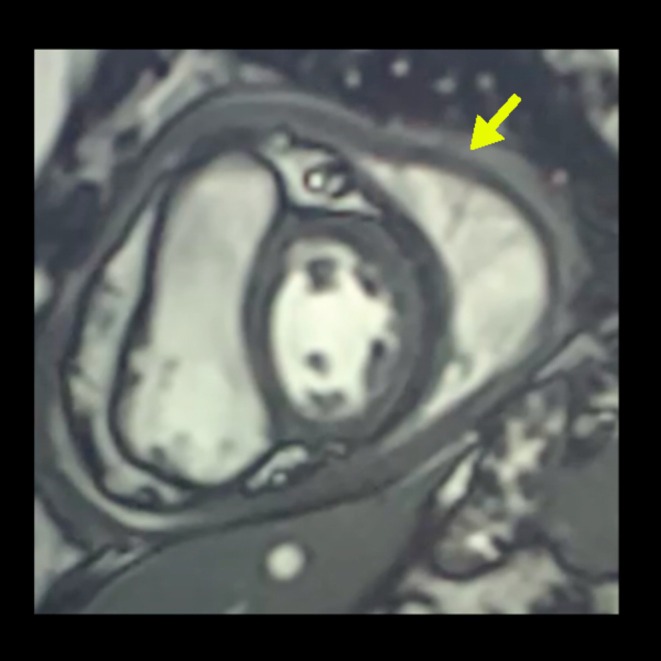
Cardiac magnetic resonance imaging in Case 2. Short‐axis black‐blood T1‐weighted post‐contrast sequence at mid‐ventricular level demonstrating severe circumferential pericardial thickening (maximum 11 mm, yellow arrow) with intense enhancement consistent with active pericarditis.

FDG PET‐CT demonstrated markedly increased metabolic activity in serosal surfaces including pericardium (SUVmax 6.8), pleura (SUVmax 5.4), and renal fascia (SUVmax 5.9), as well as diffuse uptake along vascular walls, particularly the entire aorta from arch to bifurcation (SUVmax 7.2) (Figure 5). Microbiological studies excluded bacterial, fungal, and mycobacterial infections. Comprehensive autoantibody panel including ANA, anti‐dsDNA, ANCA, anti‐Ro, anti‐La, rheumatoid factor, and IgG4 levels was negative or within normal limits. Bone marrow aspirate and biopsy showed no pathological findings.

**FIGURE 5:**
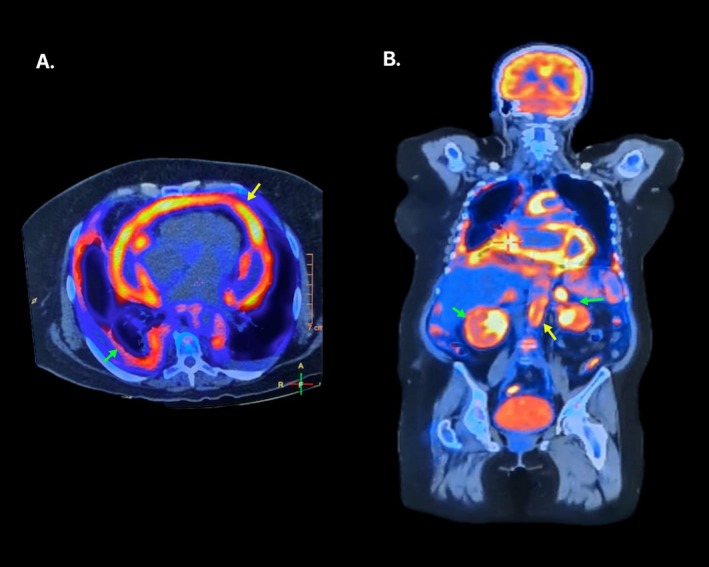
Fluorodeoxyglucose positron emission tomography‐computed tomography (FDG PET‐CT) fused images in Case 2. (A) Axial fused image at cardiac level demonstrating markedly increased metabolic activity in pericardium (SUVmax 6.8, yellow arrow) and pleura (SUVmax 5.4, green arrow). (B) Coronal fused image showing diffuse FDG avidity along the entire aortic length from arch through thoracic and abdominal segments (SUVmax 7.2, yellow arrow) and bilateral perinephric tissue (renal fascia SUVmax 5.9, green arrows), demonstrating the characteristic pattern of systemic involvement in Erdheim‐Chester disease.

Given the lack of skeletal uptake on PET‐CT, bone scintigraphy with technetium‐99 m was performed to assess for the characteristic symmetric long bone uptake of ECD. This revealed increased radiopharmaceutical uptake in the diaphysis and proximal metaphysis of the left tibia with epiphyseal sparing, a pattern highly suggestive of ECD.

The patient's symptoms intensified with progressive dyspnea and peripheral edema. Repeat echocardiography demonstrated hemodynamic features of constrictive pericarditis including respirophasic ventricular septal shift, preserved ejection fraction, and elevated filling pressures. She underwent pericardiectomy, left pleurectomy, and decortication. Intraoperative findings confirmed marked thickening of pericardial and pleural surfaces.

Histopathology of pericardial and pleural tissue showed fibrous thickening with dense collagen deposition and numerous histiocytic cells in a background of chronic inflammation. Immunohistochemistry demonstrated CD68‐positive, CD163‐positive, CD1a‐negative cells, many with a foamy appearance and several Touton‐type multinucleated giant cells, consistent with Erdheim‐Chester disease.

#### 2.2.3 Molecular Testing

BRAF V600E mutation testing was requested through polymerase chain reaction (PCR) on a peripheral blood sample and sent to a reference laboratory. However, results were not obtained before the patient's death. The absence of molecular confirmation limited the ability to pursue BRAF‐targeted therapy and precluded definitive prognostic stratification.

#### 2.2.4 Outcome

The patient was referred to hematology for systemic therapy but died from disseminated nocardiosis (confirmed by pulmonary and blood cultures with *Nocardia* species identification), likely related to immunosuppression in the context of underlying immune dysregulation from untreated ECD.

## 3 Discussion

### 3.1 Novel Presentations and Key Teaching Points

Case 1 represents one of the few reported instances of ECD presenting with predominant gastrointestinal involvement—steatorrhea, nodular gastroduodenitis, and diffuse visceral infiltration with pancytopenia and coagulopathy. Case 2 demonstrates constrictive pericarditis as an index manifestation of ECD, a presentation reported only anecdotally in the literature. Both cases illustrate critical diagnostic delays stemming from incomplete molecular profiling and highlight the imperative for rapid BRAF/MAPK testing to enable targeted therapy.

ECD is a clonal non‐Langerhans cell histiocytosis characterized by foamy histiocyte infiltration, fibrosis, and Touton‐type multinucleated giant cells, with over 50% harboring BRAF V600E mutations activating the RAS–RAF–MEK pathway [3, 4, 5, 6, 7].

Gastrointestinal involvement in ECD is exceedingly rare. Three prior cases describe similar presentations [8, 9, 10]. An Israeli report documented nodular gastritis with cobblestone pattern and patchy CD68‐positive foamy histiocyte infiltration extending from rectum to cecum [8]. Park et al. described diffuse colonic nodules with chronic non‐necrotizing granulomatous inflammation [9]. Cristophi reported bloody diarrhea with colonic ulcerations and Touton‐type giant cells [10].

Case 1 extends this phenotype with steatorrhea, hepatosplenomegaly, and autopsy‐confirmed multiorgan histiocytic infiltration (duodenum, liver, bone marrow). The constellation of gastrointestinal symptoms plus “hairy kidney” on imaging (Figure 3) should trigger ECD work‐up even without prominent skeletal involvement [11, 12, 13, 14]. FDG PET‐CT in Case 1 revealed diffuse skeletal uptake (SUVmax 8.4 femur, 7.8 spine) that was clinically silent, underscoring the value of whole‐body metabolic imaging when GI‐predominant symptoms coexist with systemic inflammation and imaging hallmarks like perinephric infiltration [15].

The pancytopenia and complex karyotype (trisomy 8, trisomy 12, Y gain) in Case 1 likely reflected myelophthisis from bone marrow infiltration (Figure 2), seen in up to 10% of ECD patients [16]. Coagulopathy may result from hepatic infiltration or disseminated intravascular coagulation. This overlap of ECD with myeloid neoplasms carrying JAK2, CALR, or cytogenetic abnormalities complicates both diagnosis and prognosis [17].

While pericardial and pleural thickening occur in up to 42% of ECD cases, constrictive physiology is rarely reported [18, 19]. Case 2 presented with progressive dyspnea, effusion, and hemodynamic constriction requiring pericardiectomy. Cardiac MRI demonstrated pericardial thickness 11 mm, respirophasic ventricular septal shift, and diffuse aortic wall thickening (8–14 mm) with intense gadolinium enhancement (Figure 4)—findings that should prompt consideration of infiltrative histiocytosis [18, 19].

The “coated aorta” sign (perivascular rind 6–12 mm) and “hairy kidney” (perinephric tissue 8–15 mm) on CT (Figure 3), combined with FDG‐avid serosa (SUVmax pericardium 6.8, aorta 7.2, renal fascia 5.9) on PET‐CT (Figure 5), distinguished ECD from IgG4‐related disease, sarcoidosis, and tuberculosis (Table 1) [13, 15]. Bone scintigraphy confirmed symmetric tibial diaphyseal uptake with epiphyseal sparing, a pathognomonic pattern absent on initial PET‐CT, emphasizing complementary roles of these modalities [20].

**TABLE 1:** Differential diagnosis of Erdheim‐Chester Disease.

Feature	ECD	IgG4‐RD	RDD	LCH	Sarcoidosis
**Imaging Hallmarks**	Symmetric long bone osteosclerosis (diaphysis/metaphysis, epiphysis spared) “Hairy kidney” sign “Coated aorta” sign (circumferential) Diffuse FDG avidity (bone, aorta, serosa)	Retroperitoneal fibrosis (spares posterior aortic wall) Pancreatic/salivary enlargement Low FDG uptake	Lymphadenopathy (massive, painless) Extranodal masses Variable FDG uptake	Lytic bone lesions (skull, ribs, vertebrae) Pulmonary cysts Pituitary stalk thickening High FDG uptake	Bilateral hilar lymphadenopathy Pulmonary nodules (perilymphatic) Low‐moderate FDG uptake
**IHC Panel**	CD68+ CD163+ Factor XIIIa+ Fascin+ CD1a− S100−/weak focal Langerin−	CD68+ CD163+ IgG4+ plasma cells > 40% IgG+ cells CD1a− S100−	CD68+ S100+ CD163+ CD1a− Langerin−	CD1a+ Langerin+ S100+ CD68+/− Birbeck granules present	CD68+ CD163+ CD1a− S100− (weak in giant cells)
**Serologies**	None specific May have ANA, anti‐dsDNA (12%–21%) Elevated CRP/ESR	Elevated serum IgG4 (> 135 mg/dL in 60%–70%) Elevated IgG Hypocomplementemia	None specific Polyclonal hypergammaglobulinemia	None specific	Elevated ACE (60%) Hypercalcemia (10%) Elevated serum lysozyme
**Clinical Clues**	Xanthelasmas Diabetes insipidus Bone pain Retroperitoneal fibrosis Cardiac/CNS involvement BRAF V600E in 50%–60%	Painless organ enlargement (pancreas, salivary glands) Retroperitoneal fibrosis Allergic history Steroid‐responsive	Massive painless lymphadenopathy Fever Anemia Extranodal masses (orbit, skin, CNS)	Children/young adults Polyuria/polydipsia (diabetes insipidus) Bone pain Seborrheic dermatitis	Young/middle‐aged adults Erythema nodosum Uveitis Cranial neuropathies Löfgren syndrome

Abbreviations: ACE, angiotensin‐converting enzyme; CNS, central nervous system; CRP, C‐reactive protein; dsDNA, double‐stranded DNA; ECD, Erdheim‐Chester disease; ESR, erythrocyte sedimentation rate; FDG, fluorodeoxyglucose; IgG4‐RD, IgG4‐related disease; IHC, immunohistochemistry; LCH, Langerhans cell histiocytosis; NA, antinuclear antibody; RDD, Rosai‐Dorfman disease.

Early recognition of constrictive physiology on cardiac MRI (pericardial thickening, ventricular interdependence, elevated filling pressures) may avoid surgery if BRAF‐targeted therapy is initiated promptly [21, 22]. Case 2's pericardiectomy preceded molecular results, and subsequent nocardiosis (likely secondary to immunosuppression compounded by untreated ECD) proved fatal.

### 3.2 Diagnostic Approach and Imaging Hallmarks

FDG PET‐CT is the most useful diagnostic tool, defining skeletal and visceral disease extent, guiding biopsy sites, and establishing baseline for treatment response [1, 15]. Cardinal imaging signs include symmetric long bone osteosclerosis [20], “hairy kidney,” “coated aorta,” and diffuse FDG avidity in bone, retroperitoneum, serosa, and vasculature [11, 13, 14, 15]. Technetium‐99 m bone scintigraphy demonstrates characteristic diaphyseal/metaphyseal uptake sparing epiphyses [20]. Cardiac MRI evaluates pericardial thickness, constriction, and myocardial infiltration [18, 19].

Histology shows xanthogranulomatous infiltrate with foamy histiocytes, Touton giant cells, and fibrosis [4]. Immunohistochemistry: CD68+, CD163+, Factor XIIIa+, Fascin+; CD1a−, CD207−, S100− (weak focal positivity may occur) [4]. Differential diagnosis includes IgG4‐RD, Rosai‐Dorfman disease, Langerhans cell histiocytosis, and sarcoidosis (Table 1) [1, 2, 3, 23].

### 3.3 Therapy

Neither patient received targeted therapy. In Case 1, BRAF testing was not requested due to rapid multisystem deterioration and death before molecular diagnostics could be completed. Case 2: BRAF V600E PCR on peripheral was sent to a reference laboratory but results were not available before death from nocardiosis. These cases underscore critical barriers: logistical delays in molecular testing, lack of point‐of‐care MAPK profiling, and diagnostic uncertainty delaying treatment escalation in resource‐limited settings. Current standard‐of‐care mandates BRAF V600E testing on all suspected ECD cases, as mutation status directly informs therapy (vemurafenib/cobimetinib for BRAF‐mutant; MEK inhibitors for BRAF‐wild‐type) [1]. Absence of molecular data in these cases precluded access to agents with 89% response rates and significantly improved survival [2].

Pegylated interferon‐alpha remains first‐line for non‐severe disease but requires dose escalation for life‐threatening manifestations (cardiac, CNS), with adverse effects limiting tolerability [1, 2]. BRAF inhibitors (vemurafenib) and MEK inhibitors (cobimetinib) achieve rapid responses but require indefinite therapy; 75% relapse within 6 months of discontinuation [1, 7]. Other agents (infliximab, anakinra, tocilizumab) show limited efficacy [5, 24, 25].

### 3.4 Clinical Decision Points

1. Gastrointestinal symptoms + “hairy kidney” → order FDG PET‐CT and BRAF testing. Steatorrhea, nodular enteritis, or unexplained visceromegaly with perinephric infiltration on CT warrants whole‐body PET and histiocytic work‐up, even without skeletal symptoms.
2. Unexplained constrictive pericarditis → obtain cardiac MRI and consider ECD. Pericardial thickening with aortic wall enhancement, “coated aorta,” or xanthelasmas should prompt PET‐CT, bone scintigraphy, and serosa biopsy with CD68/CD163 staining.
3. Suspected ECD → expedite BRAF/MAPK testing to enable targeted therapy. Point‐of‐care molecular diagnostics or send‐out PCR should occur simultaneously with biopsy processing, not after histology review, to avoid fatal delays.
4. Complementary imaging: PET‐CT for viscera, bone scintigraphy for skeletal confirmation. When PET lacks skeletal uptake but clinical suspicion persists, technetium‐99 m scintigraphy may reveal pathognomonic long bone pattern.

### 3.5 Limitations

This report has several limitations. Incomplete molecular profiling (no BRAF results in either case) prevented targeted therapy and definitive prognostic stratification. Imaging protocols were nonuniform (Case 2 had delayed bone scintigraphy). Absence of standardized response criteria and longitudinal therapy data limit generalizability. Single‐center experience in a resource‐limited setting may not reflect diagnostic timelines or molecular testing access in other regions. Future multicenter registries with protocolized imaging, mandatory MAPK profiling, and prospective treatment data are needed to establish evidence‐based guidelines.

## 4 Conclusion

We report two fatal ECD cases with unusual presentations—gastrointestinal involvement with steatorrhea and constrictive pericarditis—both featuring characteristic imaging signs (“hairy kidney,” “coated aorta,” symmetric osteosclerosis). When these imaging hallmarks accompany atypical organ manifestations, clinicians should expedite FDG PET‐CT, histopathologic confirmation with CD68/CD163 staining, and BRAF/MAPK testing to enable early targeted therapy and improve outcomes.

## Author Contributions

**Ribero‐Vargas Daniel Andres:** data curation, formal analysis, resources, supervision, validation, visualization, writing – original draft, writing – review and editing. **C. Álvarez Payares José:** conceptualization, data curation, formal analysis, investigation, methodology, project administration, supervision, visualization, writing – original draft, writing – review and editing. **Murillo‐Pérez Juliana:** conceptualization, data curation, investigation, resources, supervision, validation, visualization, writing – review and editing.

## Funding

The authors have nothing to report.

## Ethics Statement

The authors have nothing to report.

## Conflicts of Interest

The authors declare no conflicts of interest.

## Data Availability

The data supporting this study's findings are available from the corresponding author upon reasonable request.
